# Effect of Alkyl Chain Length in POSS Nanocage on Non-Isothermal Crystallization Behavior of PCL/Amino-POSS Nanocomposites

**DOI:** 10.3390/polym11101719

**Published:** 2019-10-19

**Authors:** M. Dolores Fernández, Dailyn J. Guzmán, Johnny R. Ramos, M. Jesús Fernández

**Affiliations:** Department of Polymer Science and Technology, Faculty of Chemistry, University of the Basque Country (UPV/EHU), P° Manuel Lardizábal 3, 20018 San Sebastián, Spain; mariadolores.fernandez@ehu.es (M.D.F.); daylyngm@yahoo.es (D.J.G.); johnram@hotmail.com (J.R.R.)

**Keywords:** poly(ε-caprolactone) nanocomposites, POSS nanoparticles, DSC, nonisothermal crystallization, kinetic

## Abstract

The study of the non-isothermal crystallization behavior of polymers is of great importance due to the effect of degree of crystallinity and crystallization process on the polymer properties. The effect of aminopropylisobutyl polyhedral oligomeric silsesquioxane (APIBPOSS) and aminopropylisooctyl polyhedral oligomeric silsesquioxane (APIOPOSS) on poly(*ε*-caprolactone) (PCL) crystallization is studied by differential scanning calorimetry (DSC) under non-isothermal conditions and polarized optical microscopy (POM). The crystallization kinetics is analyzed using the Avrami and Mo models, and effective activation energies are evaluated by the Friedman isoconversional method. The results show that the compatibility between polyhedral oligomeric silsesquioxanes (POSS) and PCL and POSS loading affect the crystallization process. A higher crystallization temperature, a narrower size distribution of crystallite, and a faster crystallization rate are obtained in the presence of all the studied contents of APIBPOSS and at lower contents of APIOPOSS. At APIOPOSS contents higher than 2 wt %, the crystallization temperature is lowered, the size distribution of crystallite is broadened, and the crystallization process is retarded. The presence of POSS leads to an increase in the number of nucleation sites, and a reduction in the size of the crystallite and the overall degree of crystallinity, as a result of the confinement of PCL chains caused by POSS nanoparticles.

## 1. Introduction

Poly(*ε*-caprolactone) (PCL), a semi-crystalline aliphatic and biodegradable polyester, compatible with many types of polymers and having good biocompatibility, has been used in a wide range of applications including food packaging material, tissue engineering scaffolding, medical devices, and drug delivery systems [1,2]. However, some disadvantages such as slow crystallization rate, deficiencies in thermal stability, and poor mechanical properties have limited the applications of PCL. To overcome these problems, some nanofillers have been added to PCL, and significant improvement in the physical properties of PCL can be achieved with quite a small amount of nanofillers [3,4,5,6,7].

Polyhedral oligomeric silsesquioxanes (POSS) are one of the most extensively used nanoparticles in the preparation of hybrid polymer nanocomposites. The three-dimensional POSS molecules are characterized by a cubic core (Si_8_O_12_), which is surrounded by organic groups linked to silicon atoms via covalent bonding. POSS molecules have been incorporated into organic polymers via copolymerization, grafting, or blending [8,9,10]. The incorporation of POSS molecules into some semi-crystalline polymers affects their crystallization behavior [11,12,13], and the physical and mechanical properties of a semi-crystalline polymer are strongly dependent on its crystallization and morphology.

The crystallization process can proceed under either isothermal or non-isothermal conditions. The study of the non-isothermal crystallization kinetics of polymers is important since their processing often proceeds under non-isothermal conditions. Investigation of crystallization behavior can serve as a guide for process and application.

PCL is one of the polymers that have been blended with POSS. Various POSS derivatives have been used to prepare PCL-based nanocomposites [12,13,14,15,16,17]. Pan et al. [12] studied the morphology, isothermal, and non-isothermal melt crystallization of PCL/octaisobutyl-POSS nanocomposites. They found that the crystallization of PCL is enhanced by the presence of POSS and influenced by the POSS loading. They suggested that the presence of POSS might provide heterogeneous nucleation sites for the PCL crystallization while the aggregates of POSS might restrict large crystal growth of PCL. Miltner et al. [13] investigated the influence of the addition of aminopropylheptakis(isobutyl)-POSS into PCL on the thermal properties. The results showed that POSS nanoparticles barely nucleated the PCL crystallization, and therefore hardly affected the PCL semi-crystalline morphology and thermal behavior. Goffin et al. [14] reported the crystallization behavior and the thermomechanical properties of PCL/aminopropylheptakis(isobutyl)-POSS and PCL/aminopropylheptakis(isobutyl)-POSS-g-PCL. They saw that well-dispersed POSS nanoparticles acted as efficient nucleating sites, significantly increasing the crystallinity degree of PCL. Guan and Qiu [16] studied the isothermal crystallization, morphology, and dynamic mechanical properties of PCL in the PCL/octavinyl-POSS nanocomposites. The presence of ovi-POSS enhanced the overall isothermal melt crystallization rates of PCL in the nanocomposites, while the crystallization mechanism and crystal structure of PCL remained unchanged despite the POSS loading. Lee and Chang [17] reported the effect of trisilanolphenyl-POSS on the thermal and mechanical properties of PCL/POSS nanocomposites. They found that the degree of crystallinity of the PCL decreased with incorporation of POSS into PCL. To the best of our knowledge, there are no other reports indicating that the crystallization behavior of PCL blends with different POSS derivatives having different alkyl substituents. However, it has been reported that not only the POSS content but also the structure of the alkyl chains of POSS substituent groups have an effect on the crystallization kinetics of polymer/POSS nanocomposites [11,18,19,20,21].

The purpose of the present study is to ascertain the effect of the alkyl-chain length of the nonreactive organic substituents attached to the corner silicon atoms of two amine-functionalized POSS on crystallization behavior of PCL. Recently, we have reported the effect of the alkyl-chain length of these substituents attached to the corner silicon atoms of amino-derivative POSS on morphology, thermal, and mechanical properties of PCL/amino-POSS nanocomposites [22]. We found that the longer the alkyl chain, the better the extent of dispersion of POSS due to the greater compatibility with PCL chains. In the present study, aminopropylisobutyl polyhedral oligomeric silsesquioxane (APIBPOSS) and aminopropylisooctyl polyhedral oligomeric silsesquioxane (APIOPOSS) have been used to prepare PCL/APIBPOSS and PCL/APIOPOSS nanocomposites, respectively, with different POSS contents. The effects of the incorporation of amino-POSS derivatives on the non-isothermal melt crystallization kinetics and spherulitic morphology of PCL were investigated by differential scanning calorimetry (DSC) and polarized optical microscopy (POM). We have found that the POSS structure and content had an effect on the rate of crystallization and the overall amount of crystallinity of PCL. Lower degree of crystallinity and crystallization rate have been achieved using the POSS molecule, showing better compatibility with PCL compared to the less compatible POSS. The obtained material can be of semi-crystalline nature or almost amorphous depending on the POSS loading and the alkyl-chain length of the nonreactive organic substituents attached to the vertices of the POSS cage.

## 2. Materials and Methods

### 2.1. Materials

PCL (*M*_n_ = 4.5 × 10^4^ g mol^−1^) was purchased from Aldrich (Munich, Germany). APIBPOSS and APIOPOSS were obtained from Hybrid Plastics (Hattiesburg, MS, USA), and used as received.

### 2.2. PCL/POSS Nanocomposite Preparation

Nanocomposites with 2, 5, and 10 wt % POSS were obtained via solution and casting method as described elsewhere [22]. Both PCL and POSS were first dissolved in chloroform separately. Then they were mixed together and the mixture was sonicated for 3 h. The mixture was poured into a dish to evaporate the solvent at room temperature for 24 h. The obtained film was further kept in a vacuum at 50 °C for 2 days to remove the solvent completely. For comparison, the neat PCL film was prepared in the same way. The nanocomposites were named PCL/APIBPOSS-x and PCL/APIOPOSS-x, where x denotes the weight percentage of POSS.

### 2.3. Sample Characterization

The non-isothermal crystallization characteristics of the pure PCL and PCL/POSS nanocomposites were measured with a differential scanning calorimeter (DSC-Q2000) from TA Instruments (New Castle, DE, USA). The temperature was calibrated with melting indium. All DSC measurements were performed under ultrapure nitrogen atmosphere on samples of about 5 mg, placed in aluminum pans. All specimens were first heated to 100 °C at 40 °C min^−1^, held for 5 min to erase previous thermal history and then were cooled to −50 °C at various cooling rates (ϕ) ranging from 5 to 25 °C min^−1^. After that, the samples were reheated from −50 °C to 100 °C at 10 °C min^−1^ to evaluate the melting behavior. The peak crystallization temperature (*T*_cp_) and heat of the crystallization (Δ*H*_c_) were obtained from the cooling scans after first heating, whereas the melting temperature (*T*_mp_) and the heat of fusion (Δ*H*_m_) were obtained during second heating of the samples.

XRD patterns were recorded on a Bruker D8 Advance X-ray diffractometer (Karlsruhe, Germany) with a graphite monochromator, and the Cu Kα radiation (λ = 0.154 nm) operating at 40 kV/30 mA.

The spherulitic morphology of neat PCL and the PCL/POSS nanocomposites was analyzed by polarized optical microscope (POM) on a Leitz Aristomet microscope (Wetzlar, Germany) equipped with a Mettler Toledo FP82HT hot stage controlled by a Mettler Toledo FP80 central processor (Mettler-Toledo SAE, Barcelona, Spain). The samples used for the POM analysis were placed between two glass slides and melted on the hot stage at 100 °C at 40 °C min^−1^, held there for 5 min to erase any thermal history, and then cooled to 25 °C at 2 °C min^−1^.

## 3. Results

### 3.1. Non-Isothermal Crystallization and Melting Behavior

**Figure 1:**
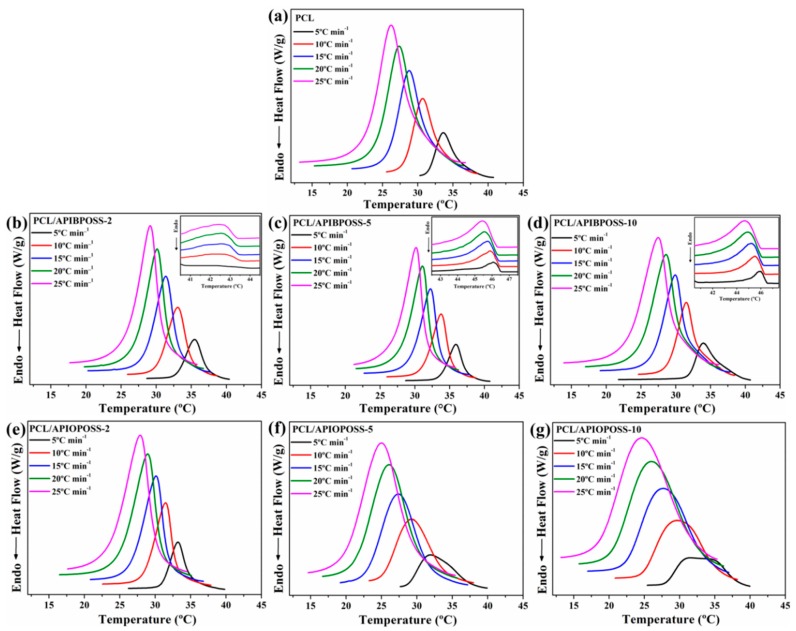
DSC traces of (**a**) PCL, (**b**–**d**) PCL/APIBPOSS nanocomposites, and (**e**–**g**) PCL/APIOPOSS nanocomposites during cooling at various cooling rates.

**Figure 2:**
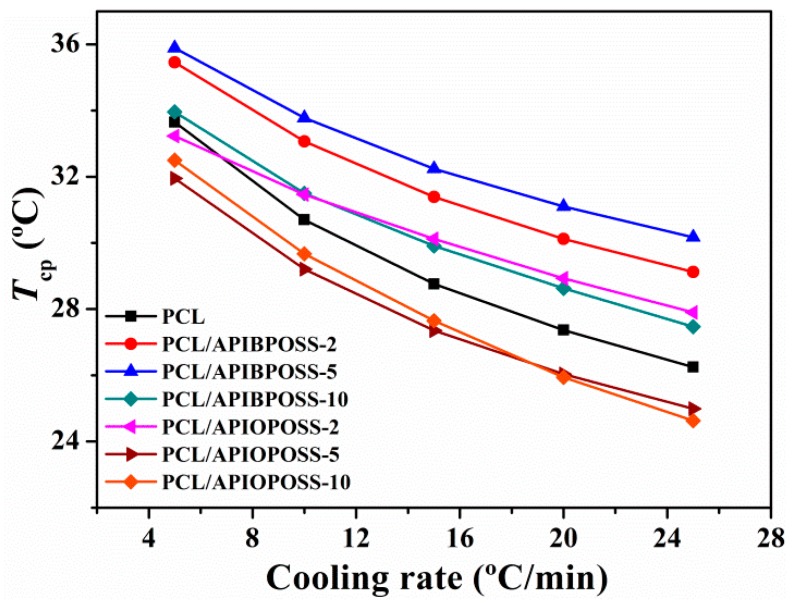
Crystallization peak temperatures as a function of cooling rate for PCL and PCL/polyhedral oligomeric silsesquioxanes (POSS) nanocomposites.

The non-isothermal crystallization thermograms at various cooling rates, ranging from 5 to 25 °C min^−1^, for neat PCL, PCL/APIBPOSS, and PCL/APIOPOSS nanocomposites are shown in Figure 1. From these crystallization exotherms, the temperature at 1% relative crystallinity (*T*_c0.01_), the temperature at 99% relative crystallinity (*T*_c0.99_), and the temperature at the maximum crystallization rate (*T*_cp_) were obtained. *T_c_*_0.01_, used to represent the beginning of the crystallization process, *T*_c0.99_, used to represent the ending of the crystallization process for PCL and nanocomposites, are reported in Table 1, while Figure 2 shows *T*_cp_ as a function of ϕ.

As the cooling rate increases, the crystallization exotherm broadens and shifts to lower temperatures for all samples, including neat PCL and POSS-based nanocomposites. With increasing of cooling rate, *T*_c0.01_, *T*_cp_, and *T*_c0.99_ shift gradually to the lower temperatures, indicating that the lower the cooling rate is, the earlier the crystallization starts. At a higher cooling rate, the activation of nuclei occurs at lower temperatures, whereas when the samples are cooled at lower scanning rates, there is enough time to activate nuclei and crystallization occurs at higher temperatures. When cooled at a low rate, the melt polymer chains have enough response time to crystallize, whereas when the samples are cooled fast, the motion of polymer molecules are not able to follow crystallization temperature.

The DSC curves of the PCL/APIBPOSS nanocomposites, in addition to the exothermic peak corresponding to the PCL crystallization, exhibit another small exothermic transition in the temperature range 41–47 °C (inset of Figure 1b–d) that corresponds to the APIBPOSS crystal formation. As we previously reported [22], APIBPOSS crystallizes and agglomerates in PCL matrix due to the POSS–POSS interactions. Due to the amorphous nature of APIOPOSS, the DSC curves of PCL/APIOPOSS composites exhibit only the exothermic crystallization peak of PCL.

The incorporation of APIBPOSS into the PCL matrix leads to an increase in *T*_cp_ of the PCL/APIBPOSS nanocomposites (Figure 2). The difference between *T*_cp_ values of neat PCL and those of APIBPOSS-based nanocomposites increases as cooling rate increases. However, the addition of APIOPOSS to the PCL matrix leads to an increase in *T*_cp_ of the nanocomposite with 2 wt % POSS loading and to a decrease in *T*_cp_ with POSS loadings greater than 2 wt %. These results suggest that APIBPOSS increases the crystallization rate of PCL, while the crystallization rate of PCL upon the addition of APIOPOSS depends on POSS loading. The nucleation of PCL crystals is retarded by the addition of APIOPOSS nanoparticles at a concentration equal or higher than 5 wt %. The addition of POSS nanoparticles as fillers into PCL shifts the crystallization peak temperature to higher values because spherulites are created around the added particles and they act as nucleation centers, making the molecular chains of PCL easier to crystallize and increasing the crystallization rate.

The total crystallization time (Δ*t*_c_) for neat PCL and the nanocomposite is calculated as

(1) $$\Delta t_{c}=\frac{T_{0.01}-T_{0.99}}{\phi }$$

where ϕ is the non-isothermal constant cooling rate, which decreases as cooling rate increases (Table 1) for both pristine PCL and PCL/POSS nanocomposites. This result implies that as the cooling rate increases, the crystallization process becomes faster and the process takes less time to complete. For example, the crystallization period of PCL sample at a cooling rate of 5 °C min^−1^ is about 1.51 min, whereas it shortens to 0.69 min when the cooling rate is increased to 20 °C min^−1^. Crystallization of PCL and its nanocomposites occurs at a higher temperature and over a larger time with decreasing cooling rate, suggesting that the crystallization is controlled by nucleation process [23]. In addition, the values of Δ*t*_c_ are reduced for the PCL/APIBPOSS nanocomposites with 2 and 5 wt % POSS as compared to the pure PCL for all cooling rates, whereas the PCL/APIOPOSS nanocomposite with 2 wt % POSS exhibits shorter crystallization time than PCL at cooling rates higher than 5 °C min^−1^. The lowered value for the Δ*t*_c_ implies that the time required for the crystallization process to be completed is somewhat lower for the nanocomposites, and suggests a decreasing time for the growth of the crystals in the presence of POSS and possibly indicates the effect of significantly increased nucleation density in the POSS nanocomposites. POSS nanoparticles can act as nucleation centers, favoring the process and increasing the speed of crystal formation.

**Figure 3:**
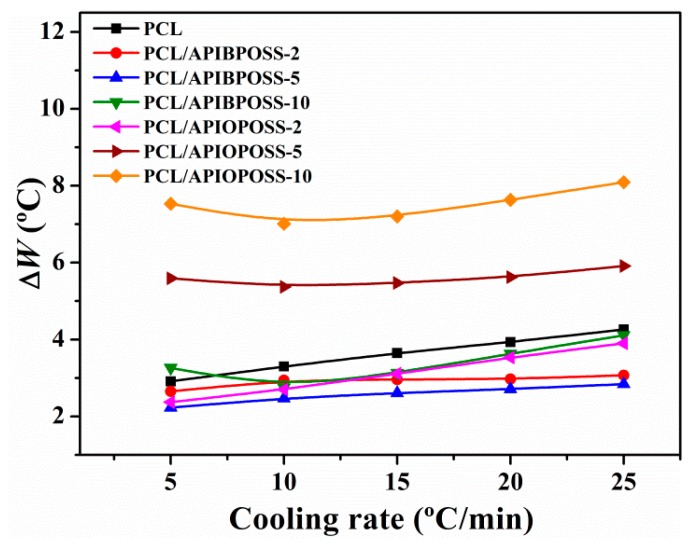
Crystallization peak width at half maximum as a function of cooling rate for PCL and PCL/POSS nanocomposites.

The width at half height of the exothermic peak of crystallization, Δ*W*, is another useful parameter to describe the non-isothermal crystallization behavior of PCL and its nanocomposites. The Δ*W* denotes the distribution of the forming crystal dimensions, that is, the smaller the Δ*W*, the narrower the distribution. It can be seen (Figure 3) that the distribution of the crystal dimensions is in the order of Δ*W*_PCL/APIBPOSS_ < Δ*W*_PCL_ < Δ*W*_PCL/APIOPOSS_ at a given cooling rate. The Δ*W* increases with the increasing cooling rate for PCL and PCL/POSS nanocomposite, which suggests that increasing supercooling results in a broader distribution of the crystal dimensions. Corresponding to the same cooling rate, the crystallite size distribution of the PCL/APIBPOSS-2 nanocomposite is the most uniform.

**Figure 4:**
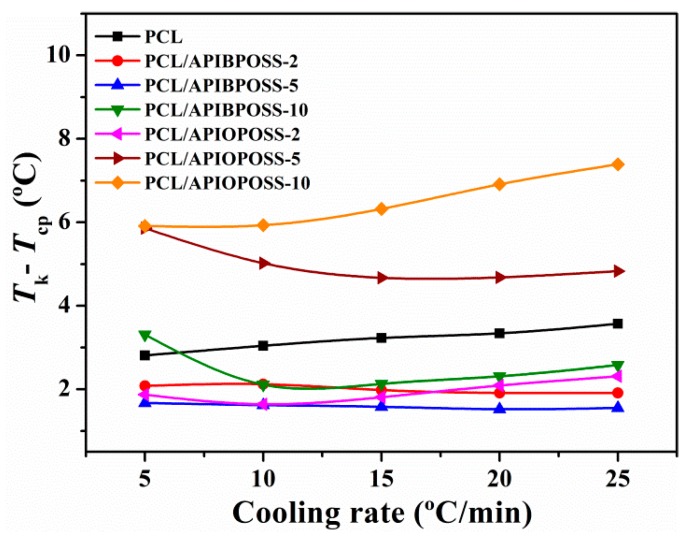
(*T*_k_ − *T*_cp_) parameter as a function of cooling rate for PCL and PCL/POSS nanocomposites.

From the DSC thermograms of the cooling process, the temperature at the intercept of the tangent at the base line and the high temperature side of the exotherm peak was obtained (*T*_k_) [24,25]. The parameter (*T*_k_ −*T*_cp_) is a measure of the overall rate of crystallization; lower values of (*T*_k_ − *T*_cp_) suggest faster crystallization. From Figure 4, it can be seen that the nanocomposites containing 5 and 10 wt % APIOPOSS exhibit the slower crystallization rate, while the nanocomposites containing APIBPOSS and 2 wt % APIOPOSS exhibit faster crystallization as compared with neat PCL.

Table 1 also shows the heat of crystallization (Δ*H*_c_). It is observed that the crystallization enthalpy is affected by both the addition of POSS and cooling rate. The values of Δ*H*_c_ obtained for both neat PCL and PCL/POSS nanocomposite decrease with increasing the cooling rate, and the addition of POSS leads to a decrease in Δ*H*_c_, indicating that the crystallinity of PCL is affected by the incorporation of POSS.

**Figure 5:**
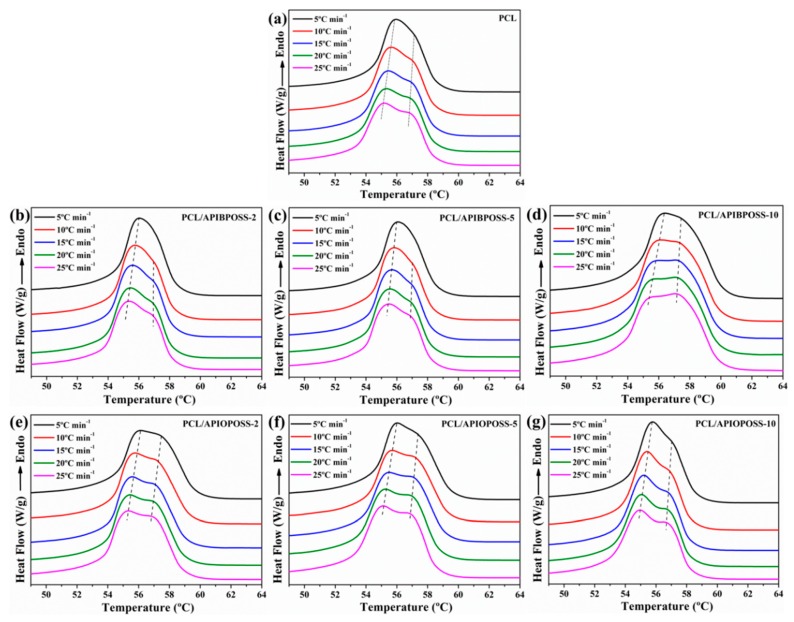
DSC thermograms of melting of (**a**) PCL, (**b**–**d**) PCL/APIBPOSS nanocomposites, and (**e**–**g**) PCL/APIOPOSS nanocomposites obtained during heating at 10 °C min^−1^ after non-isothermal crystallization at the indicated cooling rates.

The thermograms for the second heating process are shown in Figure 5. The melting temperature (*T*_mp_) and the melting enthalpy (Δ*H*_m_) were evaluated from these curves. The results of the melting behavior following each of the non-isothermal crystallization experiments are reported in Table 1.

The melting endotherm of neat PCL exhibits a shoulder on the higher temperature side, and it becomes more obvious as the cooling rate increases. Similar behavior is observed for the PCL/POSS nanocomposites. The melting peak and the shoulder at the higher temperature shift gradually toward lower temperature with increasing cooling rate, and the endothermic curves broaden. More perfect crystals are formed when the melt is cooled at low cooling rate, resulting in a higher melting peak. When the cooling rate is increased, the polymer chains could not be correspondingly rearranged into the lattice in such a short time, resulting in less perfect crystals that melt at a lower temperature. These crystals, then during heating recrystallize and reorganize into more perfect and stable crystals that melt at higher temperatures. The double endothermic peak can be attributed to the melting of the crystals that recrystallize during the heating process.

**Figure 6:**
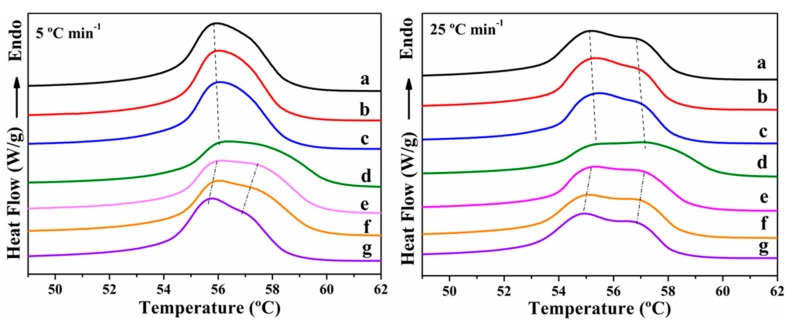
DSC thermograms of melting of (**a**) PCL, (**b**) PCL/APIBPOSS-2, (**c**) PCL/APIBPOSS-5, (**d**) PCL/APIBPOSS-10, (**e**), PCL/APIOPOSS-2 (**f**) PCL/APIOPOSS-5, and (**g**) PCL/APIOPOSS-10 nanocomposites obtained during heating at 10 °C min^−1^ after non-isothermal crystallization at the indicated cooling rates.

The *T*_mp_ of the peaks at the lower and higher temperatures (Table 1 and Figure 6) for the PCL/APIBPOSS and PCL/APIOPOSS nanocomposites are almost unchanged in comparison with the neat PCL at all cooling rates. The Δ*H*_m_ value for PCL is insensitive to the cooling rate at the studied range, whereas for PCL/APIBPOSS and PCL/APIOPOSS nanocomposites this value decreases with increasing the cooling rate, and the addition of POSS leads to a decrease in Δ*H*_m_ as compared with neat PCL.

**Figure 7:**
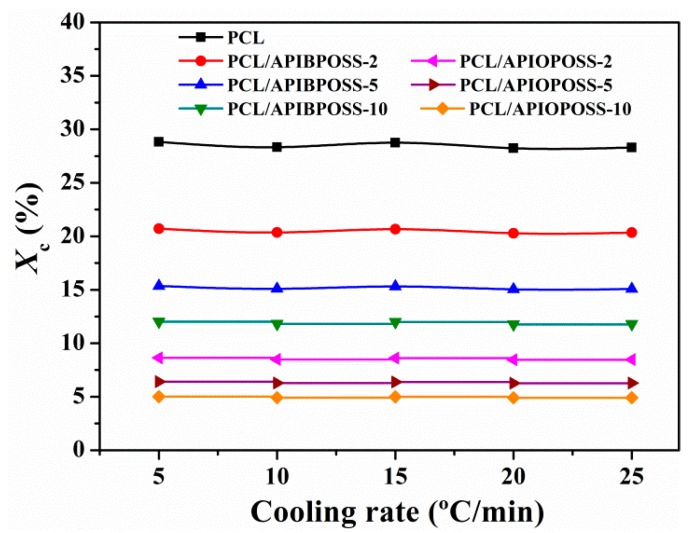
Degree of crystallinity as a function of cooling rate for PCL and PCL/POSS nanocomposites.

Figure 7 shows the extent of crystallinity (*X*_c_) calculated by the following equation:

(2) $$X_{c}=\left[\frac{\Delta H_{m}}{\Delta H_{m}^{0}\;\times \;\left(1-\frac{\%\;wt_{filler}}{100}\right)}\right]\times \;100$$

where Δ*H*_m_ is the specific heat of fusion of the sample, $\Delta H_{m}^{0}$ is the enthalpy of fusion of a perfect PCL crystal (142 J/g [26]), and % *wt_fille_*_r_ is the total weight percentage of nanofiller.

The crystallinity of PCL and its POSS-based nanocomposites are independent of the cooling rate. The addition of APIBPOSS and APIOPOSS in the PCL results in a significant reduction in the crystallinity, and the higher the POSS loading the lower the degree of crystallinity. In the case of APIBPOSS nanocomposites, at 10 wt % APIBPOSS content, the crystallinity drops from 28 to 12% as compared to neat PCL. A more pronounced reduction is observed for APIOPOSS-based nanocomposites: in the presence of 10 wt % APIOPOSS, the crystallinity falls to 5%.

The crystallinity of the polymer matrix in the presence of nanofillers can increase, decrease, or remain similar depending on the effect of the filler. If the nanofiller acts as a nucleating agent, either an enhancement or maintenance of crystallinity is expected, whereas a decrement is observed if the nanofiller hinders the mobility of the polymer chains. The decrease in crystallinity is not the expected behavior for APIBPOSS-based nanocomposites since the above results suggest that it acts as nucleating agent. The liquid APIOPOSS exhibits higher compatibility with PCL than the crystalline solid APIBPOSS, which was attributed to the more thermodynamically favorable interactions between PCL and APIOPOSS [22]. The better compatibility leads to better dispersion; hence, smaller size aggregates were found in the case of PCL/APIOPOSS blends. Crystalline aggregates of APIBPOSS molecules are present in PCL/APIBPOSS nanocomposites. The incorporation of APIOPOSS results in a retarded nucleation of PCL crystals at POSS contents higher than 2 wt %, and a remarkable reduction in the degree of crystallinity. Pracella et al. [18] reported similar results in their study of the crystallization behavior of nanocomposites of isotactic polypropylene (PP) with POSS having different alkyl substituents. They found that the liquid isooctyl-POSS displayed a very fine filler dispersion compared with the crystalline solids octamethyl-POSS and octaisobutyl-POSS, lower crystallization rate than PP and other PP/POSS nanocomposites, and no nucleation activity. They ascribed their results to the high dispersion of isooctyl-POSS as liquid phase component. Heeley et al. [20] prepared polyethylene/POSS nanocomposites bearing different long linear alkyl groups, all POSS molecules being crystalline solids. They observed that there was some aggregation of POSS crystals in the polymer matrix but, as the alkyl-chain length increased, the POSS dispersed into the amorphous domains of the polyethylene. From the crystallization behavior of their blends, they conclude that regardless of the dispersion degree in the polymer matrix, all POSS molecules acted as nucleating agents, and increased the crystallinity and crystallization kinetics when compared with pure polyethylene. Taking into account the results of our study and the studies above mentioned, it could be concluded that the liquid nature of APIOPOSS and better compatibility with PCL are responsible for the different crystallization behavior of the PCL/APIOPOSS nanocomposites compared to those containing APIBPOSS. The reduction in the overall crystallinity with increasing POSS content can be explained by the formation of larger aggregates, which hinder the transport of the polymer chains from the melt toward the crystallization growth front.

Nucleation and retarded polymer chain mobility are the two factors that influence and compete in the crystallization. The nucleation effect prevails at low amino-POSS loading, increasing the crystallization rate, whereas as the POSS content increases, the retarded mobility of the polymer chains becomes more important and dominates over the nucleation, and the rate of crystallization decreases.

Under non-isothermal conditions, the crystallization process of PCL is hindered by the incorporation of both amino-POSS derivatives, with the hindrance more pronounced in the presence of APIOPOSS.

For non-isothermal crystallization, the relative degree of crystallinity, *X_T_*, which is a function of temperature, is defined as

(3) $$X_{T}=\frac{\int_{T_{0}}^{T}\left(dH_{c}/dT\right)\;dT}{\int_{T_{0}}^{T_{\infty }}\left(dH_{c}/dT\right)\;dT}$$

where *T*_0_ and *T*_∞_ represent the initial and the end of crystallization temperature, respectively; *T* is any temperature in the crystallization process and *dH*_c_ represents the differential crystallization enthalpy change in temperature range of *dT*.

**Figure 8:**
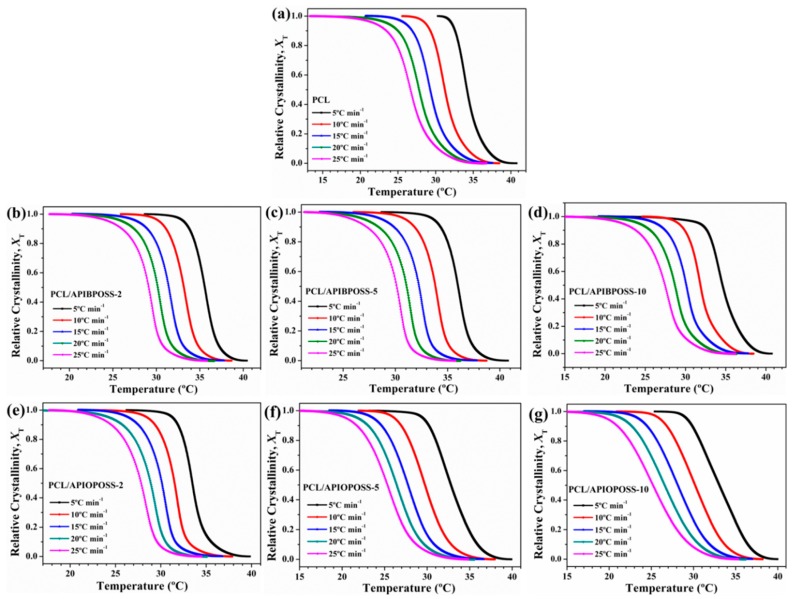
Development of relative crystallinity with temperature for non-isothermal crystallization of: (**a**) PCL, (**b**–**d**) PCL/APIBPOSS nanocomposites, (**e**–**g**) PCL/APIOPOSS nanocomposites at various cooling rates.

Figure 8 shows the relative crystallinity (*X_T_*) as a function of temperature for PCL, PCL/APIBPOSS, and PCL/APIOPOSS nanocomposites. It can be seen that all curves in Figure 8 have approximately the same reversed sigmoidal shapes. The higher the cooling rate, the lower the temperature to initiate the crystallization. There is no enough time to activate nuclei at higher temperatures when crystallized at higher cooling rates, and nucleation occurs at lower temperatures.

**Figure 9:**
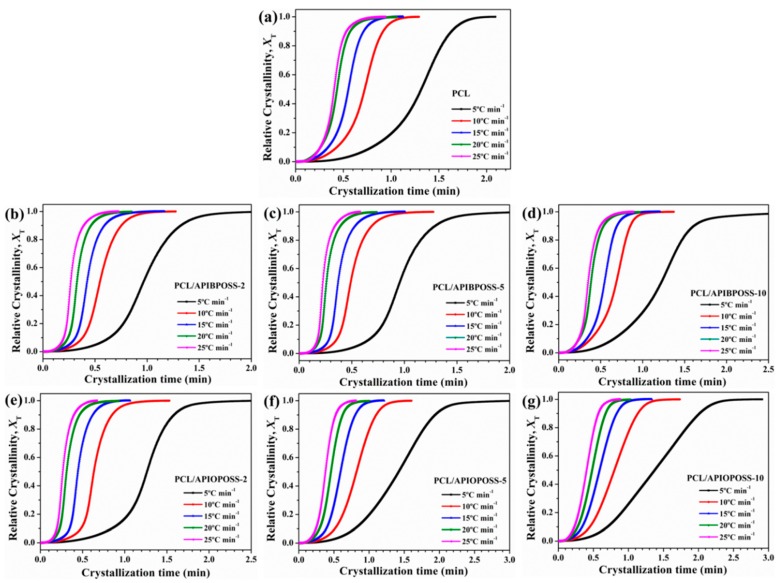
Development of relative crystallinity with time for non-isothermal crystallization of (**a**) PCL, (**b**–**d**) PCL/APIBPOSS nanocomposites, (**e**–**g**) PCL/APIOPOSS nanocomposites at various cooling rates.

The data of *X_T_* can be transformed into *X_t_* using the following equation:

(4) $$t=\frac{T_{0}-T}{\phi }$$

where *T* is the temperature at the crystallization time *t*, and ϕ is the cooling rate. The plots of *X_t_* as a function of *t* for neat PCL, PCL/APIBPOSS, and PCL/APIOPOSS nanocomposites are presented in Figure 9. All the curves have similar sigmoidal shapes, and the lower the cooling rate is, the larger the time range over which the crystallization occurs, implying that the crystallization is controlled by the nucleation process. Crystallization of PCL and PCL/POSS nanocomposites occurs at a higher temperature and is completed in a longer time under a lower cooling rate. Three distinct periods for these S-shaped crystallization curves can be observed: an induction period corresponding to the primary nucleation process that takes place in the homogeneous melt, followed by a rapid increase of the crystallization where the crystal growth occurs at the crystal-melt interface, and ultimately a constant value of crystallinity is reached because of the spherulite impingement in the later stage of crystallization. During the early stage, fast primary crystallization happened, and in the later stage slow secondary crystallization occurred. At higher cooling rates, the transition between the primary and secondary crystallization regions was less pronounced.

**Figure 10:**
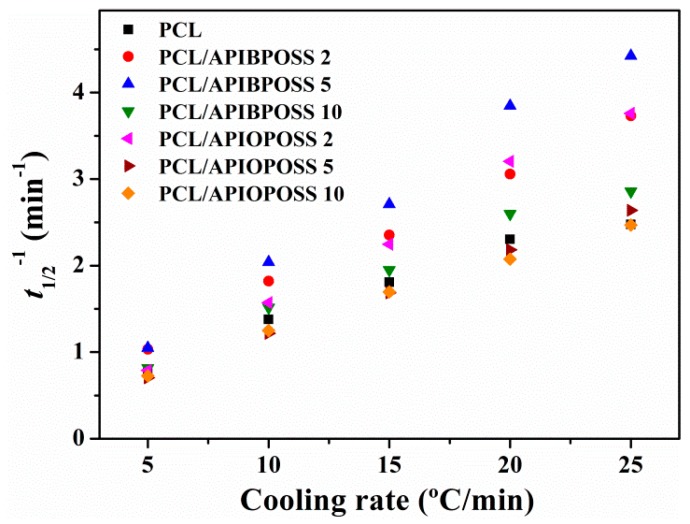
Reciprocal halftime of crystallization at different cooling rates for PCL and PCL/POSS nanocomposites.

From Figure 9, the half time of crystallization (*t_1/2_*) can be taken directly, which is the change in time from the beginning of crystallization to the time at which *X_t_* is 50%. The inverse value of *t_1/2_* denotes the bulk crystallization rate and the lower the 1/*t_1/2_* value, the slower the crystallization. Figure 10 presents the values of reciprocal of crystallization halftime as a function of cooling rate.

The 1/*t_1/2_* values depend on the cooling rate, increasing with increasing cooling rate, indicating that non-isothermal crystallization rate of PCL and nanocomposite becomes faster with increasing cooling rate. When cooling occurs at a higher rate, quick freezing of chain mobility takes place. The crystallization rate is higher for all PCL/APIBPOSS samples and PCL/APIOPOSS at 2 wt % loading than neat PCL, and lower for PCL/APIOPOSS containing 5 and 10 wt % POSS. The crystallization processes of PCL/APIBPOSS nanocomposites are finished in shorter time than that of pure PCL at cooling rates ≥ 10 °C min^−1^, while at cooling rates lower than 10 °C min^−1^, PCL and its nanocomposites exhibit similar 1/*t_1/2_* values. These results indicate that APIBPOSS nanoparticles could accelerate the crystallization process of PCL, signifying that APIBPOSS acts as nucleation agent; therefore, time needed for crystallization shortens. In the PCL/APIOPOSS nanocomposites, the sample containing 2 wt % behaves as APIBPOSS-based nanocomposites, whereas in the nanocomposites containing 5 and 10 wt % APIOPOSS, the crystallization process completion takes a longer time than that of pure PCL. The presence of APIOPOSS at 5 and 10 wt % loading obstructs the crystal growth process of PCL, hindering the crystallization of PCL.

In order to quantitatively compare non-isothermal crystallization rates obtained for neat PCL and PCL/POSS nanocomposites, two approaches can be used: (1) the crystallization rate coefficient (CRC) [27] and (2) the crystallization rate parameter (CRP) [28]. The CRC can be determined from the slope of a line by plotting the cooling rate against *T*_m_ −*T*_cp_. The higher the CRC value, the faster the crystallization rate. The CRP can be determined from the slope of a line by plotting 1/*t*_1/2_ versus cooling rate (Figure 10). The faster the crystallization rate, the higher the slope. The values or CRC and CRP are summarized in Table 1. The CRC value for PCL (3.271 min^−1^) is lower than that of PCL/POSS nanocomposites containing 2, 5, and 10 wt % APIBPOSS and 2 and 5 wt % APIOPOSS (3.806, 4.171, 3.333, 4.655, and 3.511 min^−1^, respectively), indicating that crystallization rate increases when adding APIBPOSS and APIOPOSS at these concentrations. However, the CRC value of PCL/APIOPOSS-10 nanocomposite is lower than that of PCL, indicating that crystallization rate decreases when adding 10% APIOPOSS. The CRP value for PCL (0.0854 °C^−1^) is lower than that of PCL/APIBPOSS nanocomposites (0.1260, 0.1684, and 0.1029 °C^−1^) and PCL nanocomposites containing 2 and 5 wt % APIOPOSS (0.1463 and 0.0897 °C^−1^) suggesting that these materials are more crystallizable than PCL. The PCL nanocomposite containing 5 wt % APIBPOSS exhibits the highest CRC and CRP values among the three PCL/APIBPOSS composites, indicating that this content is the most effective in accelerating the crystallization rate of PCL. The nanocomposite containing 2 wt % APIOPOSS exhibits the highest CRC and CRP values among the three PCL/APIOPOSS composites. Whereas, increasing the APIOPOSS content beyond 5 wt % retards the crystallization rate. The decrease in crystallization rate with an increase in APIOPOSS content can be attributed to the restrictions imposed by nanoparticles on the mobility of PCL chains, which interfere with the growth of crystals during the crystallization process.

### 3.2. Non-Isothermal Crystallization Kinetics

The non-isothermal crystallization kinetics analysis is of practical importance since it can reflect the crystallization behavior of polymers under processing conditions. Several models have been proposed to study the non-isothermal crystallization of polymers.

#### 3.2.1. Avrami Analysis

The Avrami model assumes that crystallization occurs under a constant temperature and expresses the relationship between *X*_t_ and crystallization time by the following equation:

(5) $$1-X_{t}=\exp(-Z_{t}\;t^{n})$$

where *Z*_t_ is the rate parameter and *n* is the Avrami exponent, which describes the nucleation (homogeneous or heterogeneous) and growth (rod, disc, sphere, sheaf, etc.) processes in non-isothermal crystallization. Equation (5) is converted to the following equation

(6) $$ln\left\{-ln\left[1-X_{t}\right]\right\}=n\ln t+\ln Z_{t}$$

In the case of non-isothermal crystallization, nucleation depends on both crystallization time and temperature; however, the Avrami model assumes that only nucleation is a function of crystallization time. To overcome this shortcoming, Jeziorny suggested that the value of rate parameter Z_t_ should be adequately corrected introducing ϕ [29]. If ϕ is assumed to be constant or approximately constant, the final form of the rate parameter (*Z*_c_) that characterizes the kinetics of non-isothermal crystallization is given as follows:

(7) $$lnZ_{c}=\frac{\ln Z_{t}}{\phi }$$

**Figure 11:**
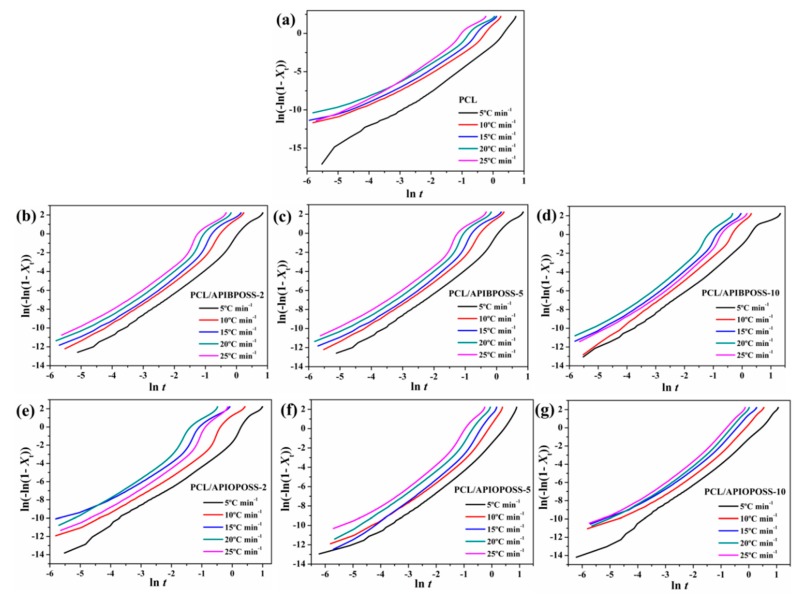
Avrami plots of ln[−ln(1−*X*_t_)] versus ln *t* for non-isothermal crystallization of (**a**) neat PCL, (**b**–**d**) PCL/APIBPOSS nanocomposites, and (**e**–**g**) PCL/APIOPOSS nanocomposites.

Drawing the straight line corresponding to ln{−ln[1 − *X*_t_]} versus ln *t*, the value of the Avrami exponent *n* and the rate parameter *Z*_t_ can be determined from the slope and the intercept, respectively. Figure 11 shows the double logarithm plots for the non-isothermal process of the PCL and PCL/POSS nanocomposites, respectively.

The curves of neat PCL and PCL/POSS nanocomposites show similar tendencies and indicate that the crystallization of the samples takes place through two main stages involving the primary and secondary crystallization processes. The primary crystallization is comprised of two different stages, indicating the complex process of the crystallization of PCL/POSS nanocomposites.

Different values of *n* and the rate parameter (*Z*_c_) for the various cooling rates and different stages of crystallization have been determined and compiled in Table 2.

It must be taken into account that in non-isothermal crystallization, the values of *Z*_c_ and *n* do not have the same respective physical meaning as in isothermal crystallization, due to the constant change of temperature under non-isothermal conditions. Nevertheless, these values can provide an insight into the kinetics of non-isothermal crystallization for PCL and its POSS-based nanocomposites. *n*_1_ and *Z*_c1_, and *n*_2_ and Z_c2_ correspond to the first stage of the primary crystallization (which corresponds to a relative conversion of 1–30%, being a little different for each curve), and to the second one (which corresponds to a relative conversion between 30 and 80%, being a little different for each curve), respectively. During the first stage of the primary crystallization, all *n* were more than 2 (Table 2), indicating that the crystallization growth likely occurred between two-dimensional and three-dimensional patterns. For PCL, all *n*_1_ values are between 2.5 and 3, and are not much affected by the presence of amino-POSS. During the second stage, the high *n*_2_ values (> 3) may be due to the combination of other complex processes. *n*_2_ values are higher than *n*_1_ values, suggesting that more complicated nucleation mechanism and geometry could be predominant as the crystallization process proceeds. In the case of PCL, *n*_2_ values are not significantly affected by the cooling rate.

#### 3.2.2. Combined Ozawa–Avrami Method

Ozawa [30] extended the Avrami equation to the non-isothermal condition by replacing the time variable by a cooling rate and derived a kinetic equation as follows:

(8) $$1-X_{t}=\exp\left[\frac{K\left(T\right)}{\phi^{m}}\right]$$

where *X*_t_ is the relative crystallinity, *K*(*T*) represents the cooling function, which is related to the overall crystallization rate and indicates the speed at which crystallization occurs, ϕ is the cooling rate, and *m* is the Ozawa exponent depending on the crystal growth and nucleation mechanism. Ozawa assumes that there is no secondary nucleation kinetics and no volume changes during the crystallization process.

**Figure 12:**
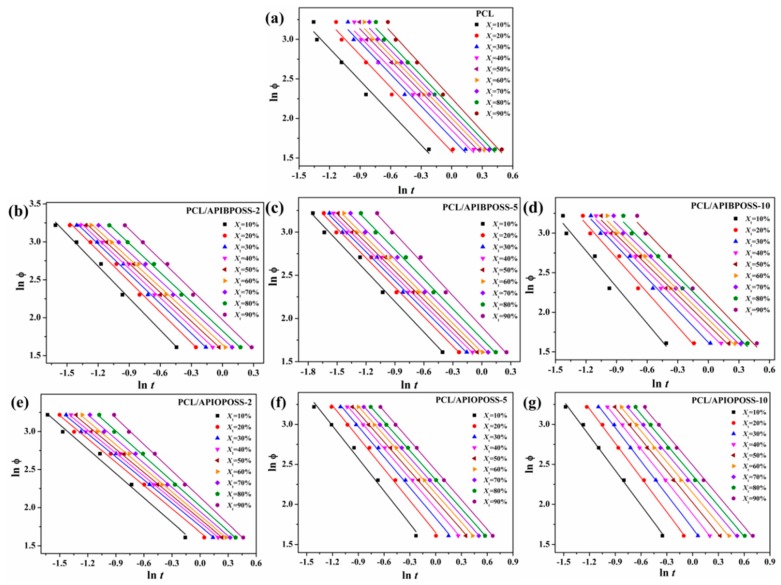
Plots of ln ϕ versus ln *t* during the non-isothermal crystallization process of (**a**) PCL, (**b**) PCL/APIBPOSS-2, (**c**) PCL/APIBPOSS-5, (**d**) PCL/APIBPOSS-10, (**e**) PCL/APIOPOSS-2, (**f**) PCL/APIOPOSS-5, and (**g**) PCL/APIOPOSS-10 at different relative degree of crystallinity.

Mo and coworkers [31] suggested a novel kinetic model by combining the Avrami Equation (5) with the Ozawa Equation (8):

(9) lnϕ=lnF(T)−αlnt

where the parameter *F*(*T*) = [*K*(*T*)/*K*]^1/m^ refers to the value of the cooling rate chosen at a unit crystallization time, when the system has a certain degree of crystallinity. The smaller the value of *F*(*T*), the higher the crystallization rate. The parameter α is the ratio of the Avrami exponent *n* to Ozawa exponent *m* (α = *n*/*m*). According to Equation (9), a straight line with an intercept of ln *F*(*T*) and a slope of –α should be obtained by plotting ln ϕ against ln *t*, at a given degree of crystallinity. As it is shown in Figure 12, plotting ln ϕ versus ln *t*, at a given degree of crystallinity, yields a good relationship. The values of *F*(*T*), α, and correlation coefficients r^2^ are listed in Table 3.

The values of *F*(*T*) increases with increasing the relative crystallinity, indicating that at unit crystallization time, a higher cooling rate should be used to obtain a higher degree of crystallinity. In addition, the value of *F*(*T*) for all PCL/APIBPOSS and PCL/APIOPOSS-2 nanocomposites are generally smaller than those for neat PCL, suggesting that crystallization rate of nanocomposites containing APIBPOSS and 2 wt % of APIOPOSS is higher than that of PCL. However, values of *F*(*T*) for PCL/APIOPOSS nanocomposites containing 5 and 10 wt % POSS are slightly higher than that of neat PCL, indicating that the crystallization rate for these nanocomposites is slower than that for PCL. These results are in good agreement with those obtained from *t_1/2_*. These results suggest that the presence of APIBPOSS nanoparticles acts as nucleating sites for the dynamic crystallization process of PCL. The values of α show a slight variation at different relative crystallinities for PCL and its nanocomposites, indicating that the method of Mo and co-workers can effectively describe the non-isothermal crystallization kinetic of PCL and PCL/POSS nanocomposites. The advantage of this method is that it correlates the cooling rate to temperature, time, and morphology, that is, nucleation and growth mechanism of crystals.

#### 3.2.3. Crystallization Activation Energy

**Figure 13:**
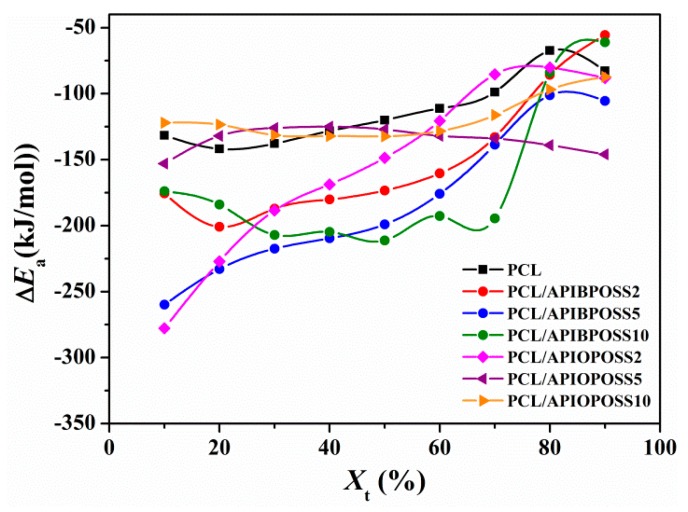
Dependence of the activation energy on the relative crystallinity using isoconversional analysis for PCL and PCL/POSS nanocomposites.

For a process that occurs on cooling, reliable values of the effective energy barrier for the process can be obtained by the differential iso-conversional method of Friedman [32]. The Friedman equation is expressed as:

(10) $$\ln{\left(\frac{dX}{dt}\right)}_{X,t}=constant-\frac{\Delta \;E_{X_{t}}}{RT_{X_{t}}}$$

where d*X*/d*t* is the instantaneous crystallization rate as a function of time at a given conversion *X*, and Δ*E_X_*_t_ is the effective energy barrier of the process at a given conversion *X*. The relative crystallinity function of temperature *X*(*T*), obtained from the experimental data shown in Figure 1, needs to be converted to the relative crystallinity function of time *X*(t) by transforming the horizontal temperature axis into time. Once the function is obtained, it is differentiated with respect to time to obtain d*X*/d*t*. Furthermore, by selecting appropriate degrees of crystallinity, the values of d*X*/d*t* at a specific *X*_t_ are correlated to the corresponding crystallization temperature at this *X*_t_, i.e., *T_Xt_*. Finally, by plotting the left hand side of Equation (10) with respect to 1/*T_Xt_*, a straight line must be obtained with a slope equal to Δ*E_X_*_t_/*R*. The dependence of the effective activation energy Δ*E*_a_ on the relative degree of crystallinity for PCL and PCL/POSS nanocomposites is shown in Figure 13.

The crystallization activation energy is closely related to crystallization process, and can exhibit the crystallization ability; high Δ*E_a_* value implies low crystallization ability. For all the samples studied, the activation energy is negative, indicating that the rate of crystallization increases with decreasing temperature. In the case of PCL, Δ*E_a_* is almost constant up to a *X*_t_ of 30%, and then increases with the degree of crystallinity up to an *X*_t_ of 80%, where the activation energy value attains a maximum value, and then decreases slightly as *X*_t_ further increases. The Δ*E_a_* values for PCL crystallization were between −140 and −67 kJ mol^−1^ in the *X*_t_ range from 10 to 90%, which are similar to those reported in the literature and obtained by the Friedman isoconversional method [33,34,35]. Δ*E_a_* value for PCL/APIBPOSS containing 2 and 5 wt % POSS increases monotonically with increasing the extent of the relative crystallization (*X*_t_), indicating that crystallization occurs much easier at lower relative crystallinity. However, the composite containing 10 wt % APIBPOSS decreases as *X*_t_ increases from 10 to 30%, then remains constant in the *X*_t_ range from 30 to 70%, and finally increases notably as *X*_t_ further increases. PCL/APIBPOSS composites show lower activation energy than that of neat PCL for a conversion range between 10 and 70%, indicating that the crystallization ability of PCL in these nanocomposites is higher than neat PCL, due to heterogeneous nucleation effect in the beginning of the process. For PCL/APIOPOSS nanocomposites, only the sample containing 2 wt % POSS exhibits lower Δ*E_a_* values than neat PCL in the relative crystallinity range from 10 to 60%, whereas activation energy values of those composites with 5 and 10 wt % APIOPOSS are lower than that of neat PCL in the conversion range from 50 to 90%. The Δ*E_a_* values of PCL/APIBPOSS composites are lower than those of the PCL/APIOPOSS composites, especially in the conversion range from 30 to 70%, indicating that the crystallization ability of PCL in the presence of APIBPOSS is higher than in the presence of APIOPOSS. At low extent of conversion (10–20%), the composites containing 2 and 10 wt % APIBPOSS exhibit higher Δ*E_a_* values than those of the composites containing 5 wt % APIBPOSS and 2 wt % APIOPOSS, which indicates that the first two need higher effective activation energy for initiation of crystallization. However, at the extent of conversion higher than 30%, PCL/APIOPOSS-2 displays Δ*E_a_* values higher than those of PCL/APIBPOSS composites, suggesting that the diffusion of the melt chains to the growth front becomes more difficult in the case of the APIOPOSS composite.

### 3.3. XRD Characterization

**Figure 14:**
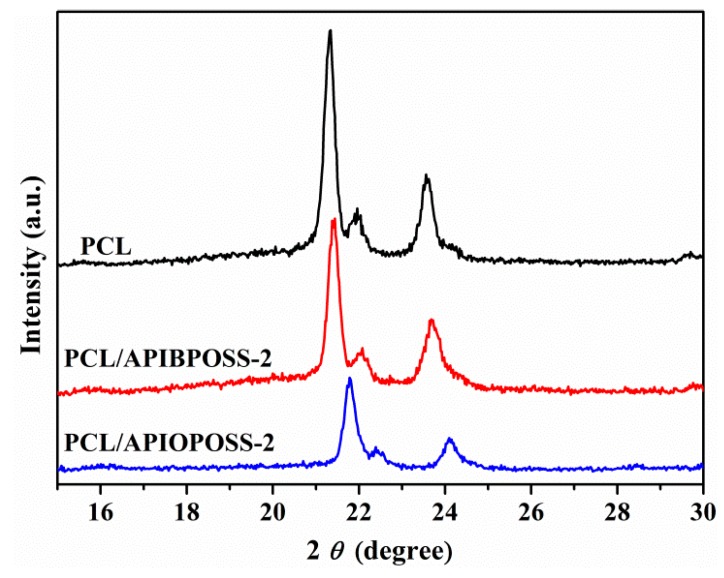
XRD patterns of neat PCL and PCL/POSS nanocomposites.

Figure 14 shows the XRD patterns of neat PCL and PCL/POSS nanocomposites containing 2 wt % POSS. Neat PCL and PCL/POSS nanocomposites exhibit the same XRD profile in the 2*θ* range of 15–30°, that is, the characteristic diffraction peaks at 2*θ* = 21.34°, 21.96°, and 23.56°. However, PCL/POSS nanocomposites show weaker diffraction peaks than PCL, and those of nanocomposite containing APIOPOSS are the weakest. This result indicates that the incorporation of POSS nanoparticles has effect on the degree of crystallinity of PCL, and this effect is more pronounced in the presence of APIOPOSS. The addition of APIBPOSS and APIOPOSS leads to a reduction in the extent of crystallinity of polymer matrix, being the reduction more remarkable when the most compatible POSS with PCL is present. This result is in agreement with the DSC results presented in this study.

### 3.4. Spherulitic Morphology

**Figure 15:**
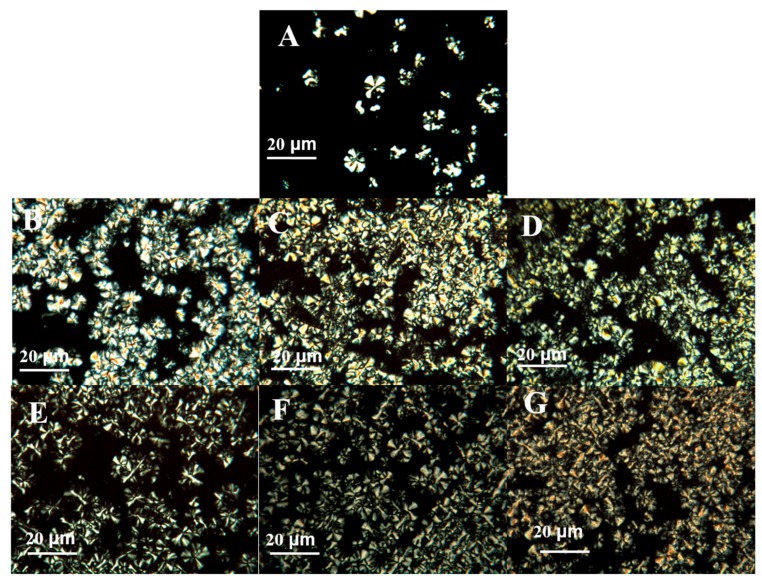
POM images of (**A**) PCL, (**B**) PCL/APIBPOSS-2, (**C**) PCL/APIBPOSS-5, (**D**) PCL/APIBPOSS-10, (**E**) PCL/APIOPOSS-2, (**F**) PCL/APIOPOSS-5, and (**G**) PCL/APIOPOSS-10 nanocomposites, at 38 °C during non-isothermal crystallization from their melts at a cooling rate of 2 °C min^−1^.

Morphology during non-isothermal crystallization was studied with a polarizing microscope. Polarized micrographs of neat PCL and its nanocomposites after non-isothermal crystallization from 100 to 25 °C at a cooling rate of 2 °C min^−1^ are shown in Figure 15. The spherulites of neat PCL are fairly larger with the maltese cross than those of the PCL/POSS nanocomposites. Compared with pure PCL, the spherulitic concentration in PCL/POSS nanocomposites is much higher, and the spherulites are smaller and more imperfect. This is attributed to the larger number of heterogeneous nuclei in PCL matrix in the presence of POSS, which reduces the sizes of spherulites.

## 4. Conclusions

Non-isothermal crystallization behavior and kinetics, and subsequent melting behavior of neat PCL, PCL/APIBPOSS, and PCL/APIOPOSS composites containing 2, 5, and 10 wt % POSS were studied by DSC. The results showed that the crystallization process was affected by the POSS type and loading. POSS hindered the crystallization of PCL due to the confinement caused by the nanoparticles on the PCL. The crystallization peak temperature of PCL shifted to higher temperature and the crystallization rate increased upon incorporation of APIBPOSS at all nanoparticle loadings studied, and at 2 wt % APIOPOSS. Based on the CRC and CRP approaches, the highest crystallization rate was obtained by addition of 5 wt % APIBPOSS and 2 wt % APIOPOSS. The Avrami and combined Ozawa–Avrami methods were able to describe the non-isothermal crystallization process of PCL/POSS nanocomposites. The activation energy for non-isothermal melt crystallization of neat PCL and PCL/POSS composites was determined using Friedman isoconversional method. The addition of APIBPOSS caused a decrease of Δ*E*. PCL/APIOPOSS composites required more energy for crystallization than PCL/APIBPOSS samples. PCL/POSS nanocomposites with 2, 5, and 10 wt % of APIBPOSS loading, and 2 wt % of APIOPOSS loading crystallized easier than PCL. Morphological analysis using POM showed that both APIBPOSS and APIOPOSS dispersed in the PCL matrix acted as a heterogeneous nucleation centers for the crystallization of PCL. The incorporation of POSS nanoparticles into the PCL matrix enhanced the nucleation density of PCL and increased with an increase in the POSS content. POSS nanoparticles played a dual role in the PCL crystallization process: first they served as a nucleating medium to promote the nucleation process of PCL, and second they hindered the mobility of PCL chains.

## Figures and Tables

**Table 1:** Differential scanning calorimetry (DSC) results of pure poly(*ε*-caprolactone) (PCL), PCL/aminopropylisooctyl polyhedral oligomeric silsesquioxanes (APIBPOSS), and PCL/aminopropylisobutyl polyhedral oligomeric silsesquioxanes (APIOPOSS).

Sample	ϕ (°C min^−1^)	*T*_c0.01_ (°C)	*T*_c0.99_ (°C)	Δ*t*_c_ (min)	Δ*H*_c_ (J g^−1^)	*T*_mp_ (°C)	*T*_mp_ − *T*_cp_ (°C)	Δ*H*_m_ (J g^−1^)	CRC (min^−1^)	CRP (°C^−1^)
PCL	5	38.92	31.38	1.508	37.34	55.96	22.31	40.94	3.270	0.0854
	10	36.70	27.38	0.932	37.20	55.65	24.95	40.22		
	15	35.41	23.86	0.770	37.45	55.45	26.69	40.82		
	20	34.33	20.43	0.695	37.43	55.34	27.97	40.09		
	25	33.73	18.35	0.615	37.45	55.20	28.55	40.20		
PCL/APIBPOSS-2	5	39.03	31.48	1.510	36.85	56.05	20.59	41.08	3.806	0.1260
	10	36.97	28.66	0.831	35.72	55.75	22.68	41.50		
	15	35.68	24.89	0.719	36.25	55.57	24.18	40.80		
	20	34.55	23.39	0.558	36.40	55.47	25.35	41.02		
	25	33.61	21.59	0.481	36.31	55.33	26.21	40.28		
PCL/APIBPOSS-5	5	39.21	31.83	1.476	35.04	56.10	20.21	39.29	4.171	0.1684
	10	37.06	29.04	0.802	35.54	55.82	22.04	37.24		
	15	35.74	26.28	0.631	35.95	55.68	23.44	37.09		
	20	34.43	24.67	0.488	35.74	55.56	24.46	37.93		
	25	33.63	23.58	0.402	35.77	55.51	25.34	36.98		
PCL/APIBPOSS-10	5	39.37	27.23	2.428	33.29	56.39	22.43	38.64	3.334	0.1029
	10	36.93	27.44	0.949	32.76	56.18	24.68	37.87		
	15	35.59	23.51	0.805	32.86	56.13	26.22	37.52		
	20	34.30	21.19	0.656	32.94	55.95	27.32	37.54		
	25	33.43	18.54	0.596	32.67	55.75	28.28	37.25		
PCL/APIOPOSS-2	5	38.13	29.52	1.722	37.60	56.13	22.90	38.46	4.655	0.1463
	10	35.84	26.29	0.955	36.91	55.74	24.28	38.62		
	15	34.44	24.14	0.687	36.81	55.6	25.48	37.95		
	20	33.03	21.26	0.588	36.63	55.43	26.50	37.96		
	25	32.21	20.56	0.466	36.41	55.34	27.44	38.36		
PCL/APIOPOSS-5	5	38.25	27.41	2.168	36.74	56.02	24.07	37.03	3.512	0.0897
	10	35.83	24.56	1.127	36.59	55.72	26.51	37.10		
	15	34.25	21.54	0.847	36.32	55.50	28.15	37.47		
	20	33.16	19.28	0.694	36.57	55.28	29.24	37.34		
	25	32.39	17.82	0.583	36.53	55.12	30.13	37.15		
PCL/APIOPOSS-10	5	38.52	28.09	2.086	36.87	55.78	23.28	37.66	3.236	0.0814
	10	36.36	24.44	1.192	36.52	55.4	25.73	37.78		
	15	34.92	21.41	0.901	36.22	55.2	27.55	38.36		
	20	33.93	19.33	0.730	36.24	55.05	29.11	37.54		
	25	32.94	17.08	0.634	35.75	54.98	30.35	37.19		

**Table 2:** Non-isothermal crystallization kinetics based on Avrami analysis for PCL and PCL/POSS nanocomposites.

Sample	ϕ (°C min^−1^)	*n*_1_	*Z*_c1_	r^2^	*n*_2_	*Z*_c2_	r^2^
PCL	5	3.06	0.735	0.999	5.38	0.694	0.999
	10	2.56	0.999	0.997	5.38	1.148	0.999
	15	2.81	1.058	0.998	5.47	1.212	0.999
	20	2.45	1.066	0.998	5.20	1.220	0.998
	25	3.07	1.081	0.999	5.36	1.213	0.999
PCL/APIBPOSS-2	5	2.67	0.805	0.995	4.67	0.950	0.997
	10	2.67	1.027	0.993	5.05	1.299	0.993
	15	2.78	1.066	0.993	6.03	1.372	0.991
	20	2.71	1.079	0.994	6.55	1.411	0.989
	25	2.57	1.073	0.993	7.02	1.425	0.993
PCL/APIBPOSS-5	5	2.94	0.803	0.995	4.54	0.966	0.993
	10	2.74	1.046	0.989	5.92	1.462	0.991
	15	2.84	1.082	0.990	7.01	1.549	0.991
	20	2.80	1.117	0.987	6.90	1.563	0.989
	25	2.88	1.110	0.988	7.60	1.551	0.989
PCL/APIBPOSS-10	5	2.48	0.624	0.997	3.95	0.804	0.995
	10	2.55	1.023	0.999	5.51	1.212	0.998
	15	2.77	1.057	0.998	6.67	1.252	0.993
	20	2.59	1.069	0.998	5.22	1.256	0.991
	25	2.68	1.061	0.993	5.67	1.247	0.993
PCL/APIOPOSS-2	5	2.45	0.688	0.996	6.27	0.690	0.998
	10	2.44	0.942	0.992	7.74	1.367	0.997
	15	2.74	1.036	0.993	7.62	1.472	0.995
	20	2.30	1.040	0.990	6.26	1.414	0.995
	25	2.43	1.055	0.992	6.15	1.364	0.997
PCL/APIOPOSS-5	5	2.55	0.711	0.995	3.40	0.738	0.999
	10	2.70	0.951	0.995	4.23	1.047	0.999
	15	2.66	0.998	0.997	4.60	1.125	0.999
	20	2.51	1.032	0.997	4.50	1.165	0.999
	25	2.62	1.047	0.998	4.59	1.163	0.999
PCL/APIOPOSS-10	5	2.67	0.770	0.998	2.69	0.785	0.999
	10	2.75	0.996	0.997	3.29	1.038	0.999
	15	2.74	1.052	0.997	3.46	1.103	1.000
	20	2.81	1.070	0.998	3.55	1.117	1.000
	25	2.75	1.076	0.998	3.47	1.118	1.000

**Table 3:** The non-isothermal crystallization kinetic parameters based on Mo model for PCL and PCL/POSS nanocomposites.

Sample	*X*_t_ (%)	10	20	30	40	50	60	70	80	90
PCL	α	1.39	1.38	1.36	1.34	1.34	1.34	1.34	1.37	1.43
	*F*(*T*)	3.39	4.77	5.68	6.30	6.85	7.32	7.81	8.42	9.42
	r^2^	0.9849	0.9899	0.9911	0.9892	0.9931	0.9932	0.9938	0.9936	0.9925
PCL/APIBPOSS-2	α	1.44	1.38	1.34	1.31	1.29	1.29	1.29	1.31	1.37
	*F*(*T*)	2.58	3.43	3.93	4.34	4.69	5.07	5.52	6.10	7.05
	r^2^	0.9958	0.9966	0.9971	0.9975	0.9978	0.9981	0.9982	0.9982	0.9967
PCL/APIBPOSS-5	α	1.18	1.13	1.12	1.11	1.11	1.12	1.13	1.16	1.23
	*F*(*T*)	3.09	3.79	4.15	4.43	4.68	4.95	5.29	5.77	6.61
	r^2^	0.9907	0.9928	0.9935	0.9938	0.9951	0.9948	0.9956	0.9962	0.9953
PCL/APIBPOSS-10	α	1.53	1.43	1.33	1.28	1.26	1.26	1.28	1.32	1.38
	*F*(*T*)	2.53	3.82	5.09	5.78	6.27	6.70	7.15	7.77	8.88
	r^2^	0.9814	0.9889	0.9933	0.9947	0.9946	0.9933	0.9927	0.9917	0.9896
PCL/APIOPOSS-2	α	1.05	1.01	1.01	1.01	1.03	1.04	1.07	1.11	1.17
	*F*(*T*)	4.54	5.42	5.82	6.09	6.34	6.62	6.98	7.49	8.39
	r^2^	0.9942	0.9961	0.9969	0.9969	0.9971	0.9969	0.9968	0.9973	0.9971
PCL/APIOPOSS-5	α	1.37	1.33	1.29	1.26	1.242	1.23	1.229	1.24	1.26
	*F*(*T*)	3.93	5.27	6.27	7.09	7.82	8.53	9.25	10.12	11.39
	r^2^	0.9916	0.9955	0.9969	0.9982	0.9982	0.9987	0.9987	0.9990	0.9990
PCL/APIOPOSS-10	α	1.38	1.39	1.37	1.34	1.32	1.29	1.28	1.28	1.29
	*F*(*T*)	3.39	4.65	5.71	6.72	7.70	8.67	9.66	10.76	13.33
	r^2^	0.9897	0.9937	0.9955	0.9967	0.9970	0.9972	0.9970	0.9970	0.9966
